# Complementary and alternative medicine use and cost in functional bowel disorders: A six month prospective study in a large HMO

**DOI:** 10.1186/1472-6882-8-46

**Published:** 2008-07-24

**Authors:** Miranda AL van Tilburg, Olafur S Palsson, Rona L Levy, Andrew D Feld, Marsha J Turner, Douglas A Drossman, William E Whitehead

**Affiliations:** 1 Center for Functional Gastrointestinal and Motility Disorders, University of North Carolina, Chapel Hill, North Carolina, USA; 2 School of Social Work, University of Washington, Seattle, Washington, USA; 3 Group Health Cooperative of Puget Sound, Seattle, Washington, USA

## Abstract

**Background:**

Functional Bowel Disorders (FBD) are chronic disorders that are difficult to treat and manage. Many patients and doctors are dissatisfied with the level of improvement in symptoms that can be achieved with standard medical care which may lead them to seek alternatives for care. There are currently no data on the types of Complementary and Alternative Medicine (CAM) used for FBDs other than Irritable Bowel Syndrome (IBS), or on the economic costs of CAM treatments. The aim of this study is to determine prevalence, types and costs of CAM in IBS, functional diarrhea, functional constipation, and functional abdominal pain.

**Methods:**

1012 Patients with FBD were recruited through a health care maintenance organization and followed for 6 months. Questionnaires were used to ascertain: Utilization and expenditures on CAM, symptom severity (IBS-SS), quality of life (IBS-QoL), psychological distress (BSI) and perceived treatment effectiveness. Costs for conventional medical care were extracted from administrative claims.

**Results:**

CAM was used by 35% of patients, at a median yearly cost of $200. The most common CAM types were ginger, massage therapy and yoga. CAM use was associated with female gender, higher education, and anxiety. Satisfaction with physician care and perceived effectiveness of prescription medication were not associated with CAM use. Physician referral to a CAM provider was uncommon but the majority of patients receiving this recommendation followed their physician's advice.

**Conclusion:**

CAM is used by one-third of FBD patients. CAM use does not seem to be driven by dissatisfaction with conventional care. Physicians should discuss CAM use and effectiveness with their patients and refer patients if appropriate.

## Background

Complementary and Alternative Medicine (CAM) therapies are growing in popularity. Between 1997 and 2002 about 35% of the US population -equaling 72 million adults- reported using CAM [1]. The use of CAM is most popular among patients who have difficult-to-treat chronic medical conditions for which conventional medicine does not provide effective treatments [2]. These patients often use CAM to augment, and sometimes even replace, conventional treatments. Functional bowel disorders (FBD) are an example of such chronic, difficult-to-treat conditions. FBD refers to a disorder of the bowels where the primary abnormality is an altered physiological function rather than a problem that has an identifiable structural or biochemical cause. FBDs cannot be diagnosed trough traditional means, such as physical examination, x-ray, laboratory tests or endoscopy. The most common FBD is Irritable Bowel Syndrome (IBS) which is characterized by abdominal pain and changes in stool consistency and/or stool frequency [3]. FBDs are common chronic disorders with approximately 30 million people in North America meeting the diagnostic criteria for IBS alone. FBDs are associated with high health care costs and health care seeking [4,5].

Since no identifiable disease processes are known to cause FBDs, treatment is focused on managing symptoms rather than affecting a cure. Many patients and doctors are dissatisfied with the level of symptom improvement that can be achieved with standard medical care [6] possibly leading many patients to seek alternative care for their symptoms. Some data are available on CAM use in Irritable Bowel Syndrome. A population-based study showed that 20.8% of IBS patients sought care from an alternative health care provider [7] and among IBS outpatients in a gastroenterology clinic in the UK, the prevalence of self-prescribed complementary and alternative oral medicines was 50.9% [8]. However, for other FBDs, such as functional constipation, there is no published data on CAM use. Although the literature is still sparse, there has been a rise in the number of studies on the effectiveness of CAM; for reviews of this literature see Tillisch [9] and Hussain & Quigley [10]. Three recent Cochrane reviews evaluate the somewhat more extensive literature on hypnosis [11], herbal medicines [12] and acupuncture [13] for the treatment of IBS, however their analyses were based on only 4 studies for hypnosis and 6 for acupuncture. Although 75 studies were included in the Cochrane review for herbal medicine, 93% of these were conducted and published in Chinese and thus unavailable to most readers outside of China.

Despite the increased acceptance and awareness of CAM, there are currently no data on the types of CAM that are used for FBDs other than IBS, or the economic cost of CAM treatments. This study aims to present data on the prevalence and different types of CAM used in IBS, functional diarrhea, functional constipation, and functional abdominal pain, as well as associated costs and perceived effectiveness. A second aim of the study is to identify patient characteristics that discriminate patients who use CAM therapies from those who do not. A third aim is to determine how often CAM therapies are recommended by physicians, and how these recommendations are perceived and acted upon by patients with FBD.

## Methods

### Setting

Data were derived from the Managing Abdominal Pain Study [14], which was carried out in 2001–2002 at Group Health Cooperative of Puget Sound, a 525,000 member health maintenance organization (HMO) in Seattle. The study surveyed patients in the care of 353 primary care physicians and 16 gastroenterology specialists in the HMO.

### Design

This study was a part of a larger investigation of standard medical care for FBDs which has been described elsewhere [5]. To identify patients with FBD, all patient encounter forms submitted by primary care and gastroenterology clinic physicians were prospectively screened for IBS (ICD-9CM code 564.1), abdominal pain (789.X), constipation (564.0), or diarrhea (787.91). This process identified patients soon after they had consulted for diagnosis and treatment of a GI complaint: This was their index visit. Patients who met inclusion criteria were mailed an invitation to participate in the study, an informed consent statement, consent to review their medical records (to exclude anyone with subsequent diagnosis of organic disease) and the first set of questionnaires, usually within two weeks of their index visit. A second set of questionnaires was sent 6 months later. Subjects were offered a $10 incentive for completing each of the surveys. This study was reviewed and approved by the institutional review boards of Group Health Cooperative, the University of Washington, and the University of North Carolina at Chapel Hill.

### CAM use and costs

Six months after the index visit, participants were asked about CAM use and cost in the previous 3 months. Recall was limited to 3 months because memory is unreliable beyond 3 months [15]. Cost was defined as out-of-pocket expenditures within the last three months, in $10 increments from $10 to $100 (the highest possible response was $100 and over). CAM therapies listed were ginger root or tea, fennel seed, senna tea, psychotherapy, homeopathic, hypnotherapy, massage therapy, biofeedback, acupuncture, yoga, aromatherapy, and evening primrose oil. Patients were also asked to write in any other alternative or home therapy they were using in addition to those listed in the questionnaire. Physician recommendation to see a CAM provider was assessed at the index visit by patient report, and percentage adherence to those recommendations (ranging from 0%–100% in 10% increments) was assessed at the six month follow-up.

In the same questionnaire, subjects were asked about non-prescription medication and supplement use including acid reducers, laxatives, anti-diarrheal medications, stool softeners, gas relief medication, pain medication, anti-spasmodics, fiber, bran, castor oil packs, enemas, suppositories, and electric heating pads or water bottles.

### Treatment effectiveness

Treatment effectiveness for prescription medications, non-prescription medications and herbal remedies was measured by self-ratings on a 5 point scale (not at all, a little, somewhat, very, and extremely). Percentage satisfaction with physician care at the index visit was recorded. Symptom improvement at 6 month follow-up was measured in two ways: (1) change in symptoms since index visit rated on a 7 point scale (markedly worse, somewhat worse, a little bit worse, no change, a little better, somewhat better, markedly better) and; (2) patients report of satisfactory relief of bowel symptoms in past 7 days (yes/no) [16].

### IBS severity and type

The Irritable Bowel Syndrome Severity Scale (IBS-SS) was used at the index visit. The IBS-SS [17] is a well-validated questionnaire for determining the overall severity of IBS symptoms. Predominant bowel activity type was determined by a single survey question: "In the last 6 months, would you describe your usual bowel movements as...?" The response options were "normal", "mostly diarrhea", "mostly constipation" or "changes back and forth".

### Psychological Distress and Quality of Life

Quality of life was assessed with the Irritable Bowel Syndrome Quality of Life Scale (IBS-QoL) [18]. This is a 34-item disease-specific quality of life measure for IBS, which has high internal consistency and reproducibility and has been shown to be responsive to changes in IBS symptom severity.

Psychological symptoms were assessed with the Brief Symptom Inventory-18 (BSI) [19]. This questionnaire quantifies the symptoms of depression, anxiety and somatization over the previous 7 days, and also provides a global severity index reflecting overall psychological distress. Due to the survey nature of this study, a slightly modified version of the BSI was used that omitted one question inquiring about suicidal thoughts, and the scores were pro-rated accordingly.

### Administrative claims

Administrative claims at the HMO were reviewed for various categories of direct costs of care for 12 months prior to the index visit and 12 months after the index visit. Additional information on the cost components can be found in a separate report on this study [5]. For each cost category, lower gastrointestinal (GI) costs were recorded separately from overall GI and non-GI costs. Health care costs were categorized as lower GI based on a list of diagnoses, drug classes, and diagnostic procedures designated by co-author Andrew Feld, MD, Chief of Gastroenterology Services for Group Health Cooperative, prior to extracting the administrative claims data. This list is available upon request.

### Data analyses

Almost all cost data deviated significantly from a normal distribution, with strong positive skew and kurtosis. Moreover, cost estimates for CAM therapies and other out-of-pocket expenditures were truncated, since the maximum reportable cost for a 3-month period was "$100 or more". In the latter case, we used $100 for this category. Medians and ranges are reported for all patients with FBD and for the subset of patients who reported using CAM therapies. Chi-square tests were used to test for differences across FBD groups in prevalence of CAM use.

Differences between CAM and non-CAM users in demographics, IBS symptoms, psychological distress and quality of life were determined by t-tests because these variables are normally distributed. Logistical regression analysis was run with CAM use as the dependent variable and all variables that showed significant univariate associations with CAM use as independent variables. Non-parametric Spearman correlations were used to determine the association of CAM costs with other health care expenditures and other patient characteristics, to address the non-normal distribution of the cost data. Alpha was .01 for all comparisons.

## Results

A total of 3024 survey packets were mailed to the selected patients. 1770 patients completed the initial questionnaires (59%) and 1012 of those completers (66%) also completed the six-month follow-up questionnaires. The analyzable sample included 419 patients diagnosed by a physician as having IBS, 183 with functional diarrhea, 159 with functional constipation, and 251 with functional abdominal pain. Table 1 shows the demographics of the complete sample.

**Table 1:** Demographics of the Sample (N = 1012)

Age	M = 53.5
	SD = 14.0
% Male	24.5%
% Hispanic	3.3%
Race	
Caucasian	89.3%
African American	2.9%
Asian	3.6%
Other	4.2%
Married/cohabiting	72.8%
College graduate	44.6%

### Complementary and Alternative Medicine Use and Cost

Of the entire sample 35.0% reported using at least one of the CAM categories. Chi-square tests did not yield significant differences across FBD groups in CAM use (see Table 2 &3). The most commonly used CAM types across all groups were ginger tea/root (13.8%), Massage Therapy (12.0%) and Yoga (10.2%). 63 Subjects reported other CAM use other than those listed of which the most common were: high fiber diet or fiber supplements (N = 7), yogurt or probiotics (N = 4), ice packs (N = 4), chiropractic treatment (N = 4) and colon cleanse (N = 3).

**Table 2:** Complementary and Alternative Medicine use and annual cost for IBS and FAP

	**IBS (N = 419)**		**Functional Abdominal Pain (N = 251)**	
		
	Use	Annual cost in $^1 ^	Use	Annual cost in $^1 ^
	%	Median(range)	%	Median(range)
Herbal Supplements/Tea				
Ginger root/tea	14.8	40 (40–320)	10.4	40 (40–240)
Evening Primrose oil	3.8	80 (40–400)	0.8	100 (80–120)
Fennel Seed Tea	4.4	40 (40–200)	1.2	40 (40-40)
Senna Tea	2.4	40 (40–360)	0.8	60 (40–80)
Massage Therapy	12.6	400 (40–400)	14.3	400 (140–400)
Yoga	10.0	80 (40–400)	5.2	80 (40–400)
Psychotherapy	8.1	280 (40–400)	2.4	100 (40–120)
Aromatherapy	7.2	40 (40–220)	5.6	120 (40–400)
Homeopathic Medications	8.1	120 (40–400)	4.4	160 (80–400)
Acupuncture	3.3	180 (40–400)	4.4	400 (40–400)
Biofeedback	1.4	40 (40–240)	0.4	120 (120–120)
Hypnosis	1.4	80 (40–400)	0.0	0 (0-0)
Any CAM	38.4	240 (40–2200)	31.9	240 (40–1280)

^1 ^Data reported for users only

**Table 3:** Complementary and Alternative Medicine use and annual cost for functional diarrhea and constipation.

	**Functional Diarrhea (N = 183)**		**Functional Constipation (N = 159)**	
		
	Use	Annual cost in $^1 ^	Use	Annual cost in $^1 ^
	%	Median(range)	%	Median(range)
Herbal Supplements/Tea				
Ginger root/tea	14.2	40 (40–200)	16.4	40 (40–320)
Evening Primrose oil	3.8	80 (40–280)	1.3	140 (120–160)
Fennel Seed Tea	2.2	40 (40-40)	0.6	40 (40-40)
Senna Tea	1.1	40 (40-40)	8.2	40 (40–240)
Massage Therapy	8.2	280 (120–400)	10.7	360 (80–400)
Yoga	8.7	80 (40–400)	6.9	40 (40–400)
Psychotherapy	2.7	120 (40–400)	2.5	380 (240–400)
Aromatherapy	3.3	40 (40–80)	5.7	120 (40–200)
Homeopathic Medications	6.0	160 (40–400)	4.4	160 (40–400)
Acupuncture	2.7	320 (160–400)	4.4	400 (200–400)
Biofeedback	0.0	0 (0-0)	0.6	40 (40-40)
Hypnosis	0.5	40 (40-40)	0.0	0 (0-0)
Any CAM	33.9	160 (40–920)	32.1	160 (40–1240)

^1 ^Data reported for users only

Among CAM users, the median cost of all CAMs combined was $200 per year, with a range from $40 to $2200. For this group the median annual cost of CAM was equal to that for their non-prescription drugs (median = $200), about two-thirds of lower GI costs (median = $308), about one-third of median pharmacy costs (median = $533), and just over 5% of total HMO health care expenses (median = $3536). Total health care expenditures and prescription drug costs were similar in CAM users compared to non-users (Table 4).

**Table 4:** Factors associated with CAM use

	CAM (N = 354)	No CAM (N = 658)	
	Mean(SD) or %	Mean(SD) or %	
Age	51.4 (13.1)	54.6 (14.3)	p < .001
Female	82.5%	71.7%	p < .001
College graduate	52.8%	40.2%	p < .001
Married/cohabiting	74.7%	69.3%	NS
			
IBS Severity (IBS-SS)	237.6 (121.4)	206.8 (113.6)	p < .001
Suffering from Distention	51.7%	40.1%	p < .001
Mainly Constipation	19.8%	18.4%	NS
Mainly Diarrhea	20.1%	19.5%	NS
			
Depression (BSI)	5.0 (4.4)	3.5 (4.8)	p < .001
Anxiety (BSI)	4.5 (5.0)	2.9 (3.7)	p < .001
Somatization (BSI)	4.6 (4.4)	3.5 (3.8)	p < .001
Quality of Life (IBSQoL)	75.1 (20.6)	81.0 (18.6)	p < .001
			
Pharmacy Costs	$1084 (1588)	$1049 (1793)	NS
Lower GI costs	$628 (1142)	$454 (522)	NS
Total health expenditures	$6059 (6628)	$6111 (8771)	NS
Non-prescription costs	$325 (369)	$192 (215)	p < .001
			
Satisfaction with physician care at index visit (0–100)	65.9 (27.1)	68.3 (25.5)	NS
Satisfactory relief of bowel Sx at follow up (% responding yes)	59.5%	63.9%	p < .01
Somewhat to remarkable change in bowel symptoms	53.7%	53.7%	NS

### Patient characteristics associated with CAM

Table 4 lists patients characteristics associated with CAM use. CAM users tended to be younger and female, have higher education, report more IBS symptom severity, less satisfactory relief of bowel symptoms, more distention, higher depression, anxiety and somatization scores, lower quality of life, and spend more on non-prescription drugs. Logistical regression analysis was run with CAM use as the dependent variable and all variables that showed significant univariate associations with CAM use as independent variables. Gender (β = -.65; p = .003), college education (β = .75, p = .000), and anxiety (β = .09, p = .008) significantly predicted CAM status (R^2 ^= .11).

### Perceived effectiveness of CAM compared to medications for FBD

Prescription medications were rated as somewhat, very or extremely effective by 68.9% (N = 358/519) of patients, compared to 61.0% (N = 356/583) for non-prescription medications and 56.4% (N = 118/209) for herbal remedies. CAM users did not rate their prescription medications as less effective (Mean = 2.9; SD = 1.1) than non-CAM users (Mean = 3.0, SD = 1.1). The median score was 3 in both groups corresponding with an answer of "somewhat effective".

### Referral to CAM providers

At the index visit, 6.4% (N = 65) of the patients were referred by their physician to a dietician, 6.6% (N = 67) to a psychologist/psychiatrist and 3.7% (N = 37) to a naturopath (naturopaths are physicians on the staff of Group Health Cooperative who have additional training in CAM therapies). Average patient confidence ratings in these recommendations were 51.3% for dieticians, 30.0% for mental health providers, and 35.9% for naturopaths. At 6 month follow up patients were asked how well the physician's instructions were followed (in increments of 10 from 0%–100%). Patients reported they had followed their physician's recommendation to see a CAM provider at least partly in 84.6% of cases for dieticians, 76.1% for psychologists or psychiatrists, and 83.8% for naturopaths.

## Discussion

### CAM Prevalence and Cost

About one third of patients who suffer from a FBD use some type of CAM for their bowel symptoms. Their median out of pocket cost for CAM is $200 per year, equaling more than one-third of pharmacy costs and two thirds of lower GI costs. Prevalence of CAM use in this study was comparable to CAM use in the general US population [1]. The most popular CAM therapies were ginger root/tea, massage therapy and yoga. The use of herbal therapies is similar to that in the US population [1] and the choice of ginger, the most popular herb, is explained by its widely suggested use for digestive problems. Several small clinical trials have supported the use of ginger as an antiemetic [20], and evidence is building for its anti-inflammatory properties [21]. The most frequently used alternative care (ginger, massage therapy and yoga) have rarely been investigated for FBDs. Hypnosis, acupuncture and peppermint oil have shown considerable benefit in a few randomized controlled trials for IBS [22,23] but are used by relatively few patients.

In the current study we defined CAM as therapies not part of conventional medicine. We chose not to include other therapies, such as fiber and probiotics, that other authors have described as CAM [8,10,24]. This decision was based on a determination that both probiotics and fiber are increasingly recommended for FBDs (both are available over the counter in the United States), and as such are more likely to be considered part of conventional medicine, rather than CAM [25,26].

### The effect of CAM on conventional care

The data suggest that using CAM does not decrease a patient's willingness to use conventional medical care. Cost of conventional care was not different between CAM and non-CAM users, CAM use was not associated with dissatisfaction with physician care at the index visit, or with perceived effectiveness of prescription medications and satisfactory relief at 6-month follow-up. However, CAM users did report more gastrointestinal symptoms at follow-up. While this association is causally ambiguous, the fact that CAM users had greater symptoms severity and greater quality of life impairment 6 months *earlier *suggests that CAM use is driven by disease severity rather than dissatisfaction with care. From the current data set we do not know why CAM use was not associated with dissatisfaction with conventional medicine. Follow-up studies are needed to study this relationship in depth.

Physicians should ask whether their patients are using CAM for at least two reasons: (1) prescribed drugs may interact with CAM or non-prescription drugs in ways that affect the safety and efficacy of the prescribed medications, and (2) physicians may wish to recommend empirically supported CAM therapies to their patients who are not using them. Koretz and Rotblatt advise in a recent review that "Gastroenterologists should become familiar with these techniques; it is likely their patients already are [27]." Referrals to CAM providers were infrequent in the current study, and confidence among patients in these treatments was low. However, physician recommendation to see a CAM provider was followed by most patients in our study, indicating a willingness of patients to consider CAM as a treatment alternative for their symptoms.

### Differences between CAM and non-CAM users

CAM use was associated with age, gender, education level, IBS severity, less satisfactory relief of symptoms, abdominal distention, psychological distress, and non prescription drug costs. Of these, only gender, college education and anxiety remained significant in a multivariate analysis. The association with psychological distress indicates a possible role for poor coping with symptoms in seeking alternative care. This finding is in contrast with the observations of Koloski and colleagues [7] who found that seeking care from an alternative health care provider was not related to psychological morbidity. The current study has focused on the use of a wide range of CAM -including self prescribed remedies- not only self-referral to a CAM provider which may explain the different findings.

### Limitations of the study

This study was limited by recruiting among HMO patients in an urbanized area in Western US. The findings may therefore have limited generalizability to uninsured patients, Medicare patients, or patients in non-urban areas. However, the demographics of the HMO membership are largely similar to that of the Unites States. In addition, any survey has the potential weakness of self-selection bias by people with certain characteristics. As we have no data on non-responders we cannot exclude self-selection bias. However, our response rate was well within the acceptable limits for medical research (59%), and CAM use was not the sole or main focus of the study and thus it is unlikely that patients self-selected on this criterion.

Secondly, patients did not specify if they used the CAM therapies for their bowel problems, to treat other symptoms, or simply to increase quality of life. The associations between CAM use and bowel disorders are therefore tentative. Additional limitations are the dependence on self-report for CAM and non-prescription cost data, and the categorical nature and ceiling limitation of its reporting. As a consequence, real CAM costs may be higher than reported in this study.

## Conclusion

In conclusion, CAM use is common in patients with FBD, but does not appear to be driven by perceived ineffectiveness of conventional medicine. Not all patients may be able to afford CAM due to its out-of-pocket costs. By moving CAM therapies with proven effectiveness into regular care, and providing insurance reimbursement for them, CAM can become more widely available. For this to occur we need to fill current knowledge gaps about treatment effectiveness (such as in herbal therapies) and make treatments already known to be effective, such as hypnotherapy and psychotherapy, more readily accessible.

## Abbreviations

CAM: Complementary and Alternative Medicine; FBD: Functional Bowel Disorder; IBS: Irritable Bowel Syndrome.

## Competing interests

The study was funded in part through a grant from Novartis Pharmaceuticals.

## Authors' contributions

All authors except MALvT contributed to study design and data collection. MALvT, OSP and WEW were involved in drafting the analysis plan and interpretation of the data. MALvT analyzed the data and was principal author of the manuscript. All authors contributed to manuscript drafting and revision and approved the final manuscript.

## Pre-publication history

The pre-publication history for this paper can be accessed here:


